# Serum Metabolic Profiling Reveals the Antidepressive Effects of the Total Iridoids of *Valeriana jatamansi* Jones on Chronic Unpredictable Mild Stress Mice

**DOI:** 10.3389/fphar.2020.00338

**Published:** 2020-03-20

**Authors:** Yongbiao Li, Lanlan Wu, Chang Chen, Liwen Wang, Cong Guo, Xiaoqin Zhao, Tingting Zhao, Xinyi Wang, An Liu, Zhiyong Yan

**Affiliations:** ^1^School of Life Science and Engineering, Southwest Jiao Tong University, Chengdu, China; ^2^Institute of Chinese Materia Medica, China Academy of Chinese Medical Sciences, Beijing, China

**Keywords:** *Valeriana jatamansi* Jones, iridoids, depression, ^1^H NMR, metabolomics

## Abstract

**Background:**

Depression is a long-term complex psychiatric disorder, and its etiology remains largely unknown. *Valeriana jatamansi* Jones ex Roxb (V. *jatamansi)* is used in the clinic for the treatment of depression, but there are insufficient reports of its antidepressive mechanisms and a poor understanding of its endogenous substance-related metabolism. The objective of this study was to identify biomarkers related to depression in serum samples and evaluate the antidepressive effects of the iridoid-rich fraction of V. *jatamansi* (IRFV) in a chronic unpredictable mild stress (CUMS) mouse model.

**Methods:**

Here, CUMS was used to establish a mouse model of depression. Behavioral and biochemical indicators were investigated to evaluate the pharmacodynamic effects. A comprehensive serum metabolomics study by nuclear magnetic resonance (NMR) approach was applied to investigate the pharmacological mechanism of IRFV in CUMS mouse. Subsequently, we used multivariate statistical analysis to identify metabolic markers, such as principal component analysis (PCA) and orthogonal projection to latent structure with discriminant analysis (OPLS-DA), to distinguish between the CUMS mouse and the control group.

**Results:**

After IRFV treatment, the immobility time, sucrose preference, and monoamine neurotransmitter were improved. PCA scores showed clear differences in metabolism between the CUMS group and control group. The PLS-DA or OPLS-DA model exhibited 26 metabolites as biomarkers to distinguish between the CUMS mice and the control mouse. Moreover, IRFV could significantly return 21 metabolites to normal levels.

**Conclusion:**

The results confirmed that IRFV exerted an antidepressive effect by regulating multiple metabolic pathways, including the tricarboxylic acid cycle, the synthesis of neurotransmitters, and amino acid metabolism. These findings provide insights into the antidepressive mechanisms of IRFV.

## Introduction

Depression, a common mental illness characterized by sadness, severely affects a patient's quality of life (Gong et al., 2019). Patients with depression exhibit symptoms including unhappiness, insomnia, anorexia, loss of interest, low mood, and anxiety (Avenevoli et al., 2015). The etiology of depression is assumed to be associated with genetics, the noradrenergic, dopaminergic, and serotoninergic systems, and stress (Drago et al., 2011). In recent years, one suffering from depression would have been primarily treated by synthetic antidepressants with emerging adverse effects, such ascardiotoxicity, hypertensive crisis, and sleep disorders (Read et al., 2014). Thus, greater therapeutic efficacy and fewer adverse effects are required. Traditional Chinese medicine (TCM) has been treating depression for more than 2,000 years. Its toxicity, safety, effectiveness, and multitargeted characteristics have received widespread attention from scholars both here and abroad in the field of depression treatment research (Yeung et al., 2012; Zhang and Cheng, 2019)

*Valeriana jatamansi* (*V. jatamansi*), originating from the herbs of Diannan written by Lan Mao (1396–1476) (Zhang et al., 2018), is a famous TCM used for relieving malaise, neurovegetation, and insomnia for more than 2,000 years (Chen et al., 2003; Wang et al., 2012). In recent years, studies have reported that *V. jatamansi* has signiﬁcant antidepressive effects (Sah et al., 2011). Tagara, in which *V. jatamansi* is used as the key ingredient for the treatment of depression-type insomnia, has been marketed abroad (Singhal and Neetu, 2013). *V. jatamansi* contains IRFV, flavonoids, alkaloids, and volatile oils (Toolika et al., 2015). IRFV is the primary sedative and active component of *V. jatamansi*. In addition, IRFV exhibits potential therapeutic effects on Parkinson's disease, anxiety, and Alzheimer's disease (Jugran et al., 2019). However, there are few reports on the antidepressive effects and mechanism of IRFV, and there is a poor understanding of the endogenous substance-related metabolism in the IRFV antidepressive state.

Metabolomics is interrelated with disease phenotype (Gao et al., 2019). Metabolomics is a nonselective and universally applicable comprehensive analytical method for the qualitative and quantitative analyses of metabolites (Zheng et al., 2011; Wolfender et al., 2013). The occurrence and development of any disease will affect the metabolism of the body. Using a metabolomics approach, biomarkers of depression can be found, which can also describe metabolic abnormalities in the pathological process of depression. NMR-based metabolomics is widely used to analyze natural extracts (Choi et al., 2006; Wolfender et al., 2015). Hence, a ^1^H NMR integrated metabolomic approach was applied to screen and identify biomarkers associated with CUMS mice and understand the antidepressive influences of IRFV in a depressive mouse model. The primary goal of this work was to elucidate the antidepressive effects of IRFV on metabolic profiles, which can be used to better understand the underlying mechanism of treating depression.

In this study, an integrated ^1^H NMR-based metabolomic approach was applied to screen and identify biomarkers associated with CUMS mice and understand the antidepressive influences of IRFV in a depressive mouse model. The primary goal of this work was to elucidate the antidepressive effects of IRFV on metabolic profiles in order to better understand the underlying mechanism of treating depression.

## Materials and Methods

### Reagents

The roots and rhizomes of *V. jatamansi* were purchased from the Lotus Pond Chinese herbal medicine market in Chengdu, Southwest China. The sample was identified by Professor Liangke Song according to the pharmacognostic standard documented in the Chinese Pharmacopoeia. A voucher specimen (No. 20181003) was deposited in the herbarium of the Laboratory, School of Life Science and Engineering, Southwest Jiaotong University, China. Fluoxetine (Flu) was purchased from Suzhou Lilai Pharmaceuticals Co., Ltd. (Chengdu, China).

A phosphate buffer solution (0.1 M, K_2_HPO_4_/NaH_2_PO_4_, pH 7.4) was prepared as the extraction solvent, which contained 10% D_2_O (99.9% D) to provide a ﬁeld lock for the NMR spectrometer. Distilled water was used for the preparation of all solutions.

### IRFV Preparation

According to the previous extraction preparation process in our laboratory (Ke-Ke et al., 2014), a total of 10.4 kg of *V. jatamansi* dry plant rhizome powdered material was soaked in 70% aqueous ethanol at room temperature with occasional shaking and extracted three times for 24 h each time. The extracts were ﬁltered through a muslin cloth and then acquired from the ﬁltrate by reduced pressure evaporation at 45°C to yield the ethanol extract (2.32 kg) and underwent ethanol–water gradient elution using a D101 macroporous resin column, first eluted with pure water and 60% ethanol. Then, the eluate was eluted with 95% ethanol, and the eluent was collected. The ethanol was concentrated and recovered under vacuum to obtain the extract (IRFV) (0.19 kg; purity: 76.58%). Furthermore, our group established a quality control standard for IRFV, and the chlorovaltrate content was 8.91 mg/g (Zhu et al., 2016). IRFV was preserved at 4°C without light.

### Experiment Animals and Drug Administration

Kunming (KM) mice (6 weeks old; weighing 20–24 g), with certificate number: SCXK (Chuan) 2015-030, were purchased from Dashuo Biological Technology Company in Chengdu and fed in the animal house of the pharmacological lab of Southwestern Jiao tong University. The mice were housed in propylene cages for an acclimatization period of 7 days prior to the experiments under controlled laboratory conditions with temperature of 25 ± 2°C, a relative humidity of 45 ± 15%, and a 12 h light/dark cycle. All animals were provided food and water. All mice were randomly divided into 6 groups (n = 7): the control group, CUMS model group, positive control group (Flu), and low, middle, and high dose IRFV treatment groups (IRFV-L, IRFV-M, IRFV-H, respectively). The experiments were approved by the Animal Ethics Committee of Southwest Jiaotong University (March 19, 2018, No. S20190319002).

Mice from the three IRFV treatment groups (IRFV-L, IRFV-M, IRFV-H) were orally administered 5.73, 11.47, and 22.94 mg/kg IRFV. Flu (2.5 mg/kg) was administered to mice in the positive control group. Mice in the control and model groups were fed an equal volume of 5% carboxymethylcellulose sodium water solution (CMC-Na, Chengdu Kelong Chemical Reagent Factory, China). All of the drugs were administered 30 min before stress exposure.

CUMS is the most extensively validated antidepressive screening (Willner, 2017) model and was used in this experiment. Mice were subjected to constant exposure to varying mild stressors for 4–8 weeks, resulting in a loss of responsiveness to rewards. The following stressors were conducted in a random order once a day for four weeks without repeating the stressors for two consecutive days: fed in individual cages including 24 h of food or water deprivation, damp sawdust for 24 h (50 ml of water per individual cage, which is enough to make the sawdust bed-ding wet), swimming in cold water at 4–8°C for 5 min, tail clamp for 1 min, and constraint for 2 h. The CUMS procedure was conducted for four weeks on all of the animals except for the mice in the control group.

### Behavioral Tests and Biochemical Indicator Test

After two weeks of drug administration, the tail suspension test (TST) and sucrose consumption test (SPT) were performed as previously described (Klein et al., 2015). In the TST, the mice were suspended individually using a suspended tail instrument (Chengdu Taimeng Software Co. Ltd). After a period of struggle, the mice would appear to tail on the instrument without struggling. This period of time was considered the immobility time. The test was performed for a total of 6 min, and the immobility time during the last 4 min was recorded using a video tracking system. In the SPT, two bottles with a 1% sucrose solution were placed in each cage for the first 24 h. Then, one bottle was replaced with a bottle of tap water for the next 24 h. The mice were deprived of water and food for 23 h and then given a bottle with 100 ml of a 1% sucrose solution and another bottle with 100 ml of tap water. The percent volume of sucrose solution consumed was calculated (sucrose preference (%) = sucrose consumption/(water consumption + sucrose consumption) × 100%). The mice were sacrificed after the behavioral tests and the hippocampal tissues were collected for homogenization. The hippocampal tissue homogenate was centrifuged (3,000 g, 5 min, 4°C). Moreover, the concentrations of corticotropin releasing factor (CRF), 5-hydroxytryptamine (5-HT) and noradrenalin (NE) in hippocampal tissues were determined using enzyme-linked immunosorbent assay (ELISA) kits.

### Sample Collection and Preparation for NMR Analysis

The whole blood of all mice was collected from the orbit, and the serum samples were acquired by centrifugation at 3,500 g and 4°C for 15 min, quickly frozen in liquid nitrogen and then stored at −80°C for further analysis. A volume of 200 μl of serum was mixed with 400 μl of a 90 mM phosphate buffer (NaH_2_PO_4_ and K_2_HPO_4_, pH 7.4) in 0.9% saline solution (100% D_2_O) and then centrifuged at 13,000 g and 4°C for 15 min. The supernatants were transferred into a 5 mm NMR tube for NMR analysis.

The serum samples were analyzed at 298 K using a Varian VNMRS 600 MHz NMR spectrometer operating at 25°C by using the Carr–Purcell–Meiboom–Gill (CPMG) spin-echo pulse sequence, where a total spin–spin relaxation delay 2 (nτ) of 320 ms was applied to attenuate the broad NMR signals from the slowly tumbling proteins and lipoproteins due to their long transverse relaxation time. The free induction decays (FIDs) were collected with 64 k data points with a spectral width of 12,000 Hz and 128 scans. The FIDs were zero-ﬁlled to double size and multiplied by an exponential line-broadening factor of 1.0 Hz before Fourier transformation (FT). In addition, standard COSY, TOCSY, HMBC and J-resolved spectra were acquired for metabolite identification purposes for the selected plasma samples.

### Multivariate Data Analysis

All of the ^1^H NMR spectra were manually phased and corrected for baseline distortion by MestReNova 7.1.0 software (Mestrelab Research, Spain). All the spectra were referenced to the methyl group of creatinine at *δ* 3.039. In order to exploit all metabolites in formation embedded in the spectra, all NMR spectra (0.5–9.0) were segmented into equal widths of both 0.01 ppm and 0.002 ppm. Spectral regions of *δ* 4.68–5.20, *δ* 3.35–3.38, and *δ* 2.06–2.08 were excluded to eliminate variations caused by imperfect water suppression, methyl alcohol, and n-acetylcysteine. The centralized data were imported into SIMCA-P 12.0 for multivariate data analysis. Principal component analysis (PCA) was applied to detect group clustering and identify outliers. Additionally, an orthogonal partial least squares discriminate analysis (OPLS-DA) algorithm was further constructed using unit-variance scaling with a seven-fold internal cross-validation and CV-ANOVA approach (*p* < 0.05). Color-coded loading plots with their absolute value of coefficients (r) were generated with MATLAB (http://www.mathworks.com) with some in-house modifications, which were performed to identify significantly altered metabolites. The main parameters of the verification model (Q2 and R2) were calculated on SIMCA-P 12.0. R2 indicates the goodness of fit of the model, whereas Q2 estimates its prediction ability (Liu et al., 2012). The variable importance in the projection (VIP) values, which expresses the significance in discriminating between groups, was used to select the biomarkers. In this study, the data with VIP > 1 were selected for independent samples, and a t-test using SPSS Statistics Base 17.0 (SPSS Inc., USA) was used to compare significant differences between two groups. Fold change (FC) was calculated as the average peak area between the two groups. After that, P values were obtained for the analysis of data statistics and the determination of the metabolites in the next step. The data with *p* < 0.05 were considered important, and data with *p* < 0.01 were considered to be very significant. The final data to identify the biomarkers were those with a VIP value greater than 1 and a P value less than 0.05 (Xiong et al., 2016). The spectra were compared with the Human Metabolome Database (HMDB) (http://www.hmdb.ca/) and related literature to finalize the metabolite species. After that, metabolites and their metabolic pathways were studied by searching the KEGG database (https://www.kegg.jp/kegg/pathway.html). The metabolite-correlation network and metabolic networks were constructed using Cytoscape software (v.3.6.) and Gene Cards (http://www.genecards.org), which were matched with the genes from the disturbed metabolic pathways (Zhou et al., 2016).

## Results

### Body Weight

**Figure 1:**
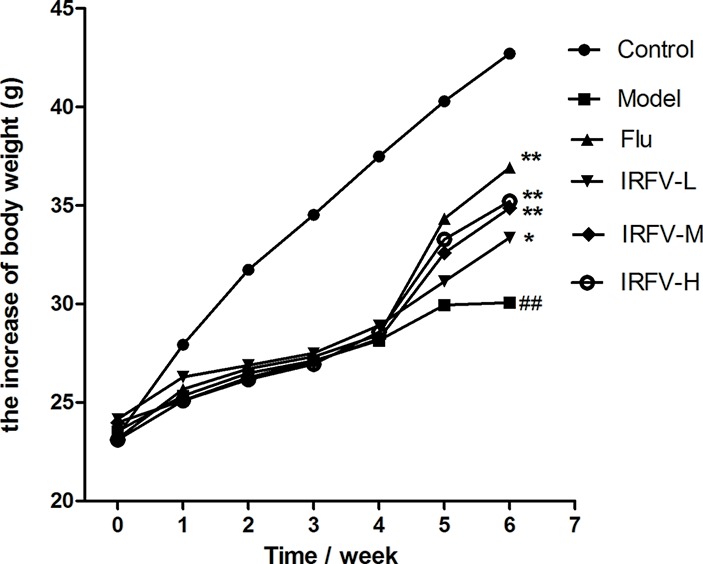
The 1^st^, 2^nd^, 3^rd^, and 4^th^ weeks after CUMS intervention and two weeks after drug administration, all mice treated with CUMS weighed slightly less than the mice in the control group. During the CUMS intervention period, there was no significant difference in body weight between the different groups (p > 0.05). During the dosing period, the mouse body weights of the control group showed a natural increase, while the body weights of the IRFV dose and Flu groups significantly increased compared to the model group (p < 0.05 or p < 0.01). However, no significant difference was observed among the weights of the different treatment groups (*p* > 0.05). ^##^p < 0.01 vs the control group; *p < 0.05, **p < 0.01 vs the model group.

The statistical analysis of body weight of mice is shown in (**Figure 1**). The 1^st^, 2^nd^, 3^rd^, and 4^th^ weeks after CUMS intervention and two weeks after drug administration, the body weights of all mice treated with CUMS were slightly lower than the mice in the control group. During the CUMS intervention period, there was no significant difference in weight between each group (*p* > 0.05). During the dosing period, the body weights in the control group showed a natural increase, while the body weights in the IRFV dosage and Flu groups significantly increased compared to the model group (*p* < 0.05 or *p* < 0.01). However, no significant difference was observed for the weights of the different treatment groups (*p* > 0.05).

### Effect of IRFV on Behavior

**Figure 2:**
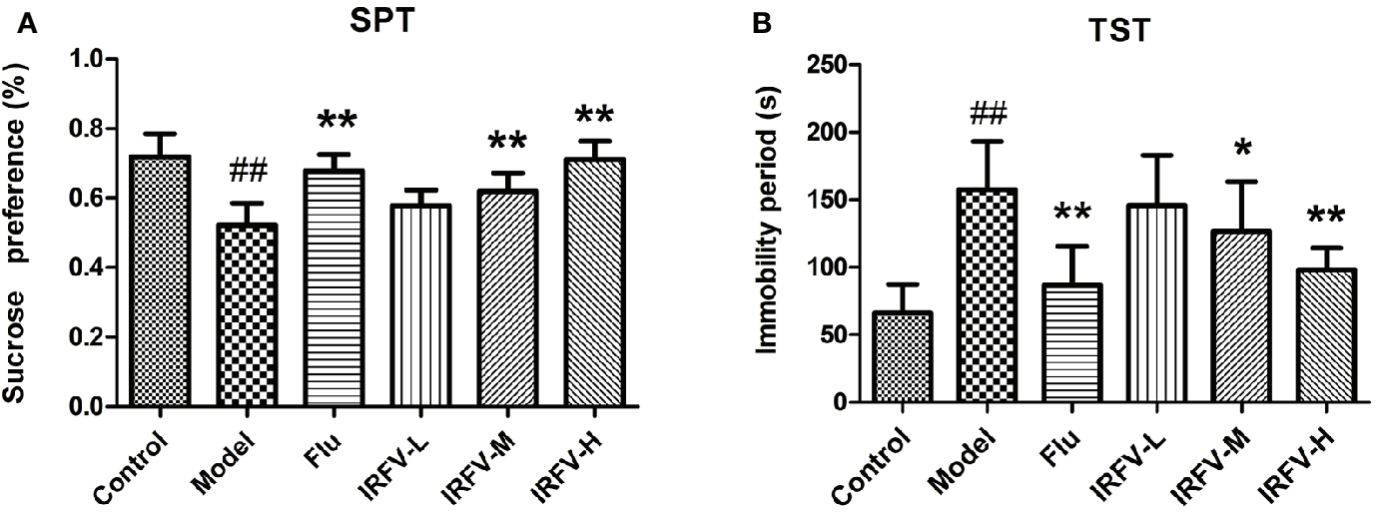
The immobility time of the TST in all groups **(A)**. In the model group, the immobility times were dramatically longer than those in the control group (*p* < 0.01). However, after administration of Flu and IRFV, the immobility time was shortened significantly (*p* < 0.05 or *p* < 0.01), but no obvious difference was observed in the IRFV-L group (*p* > 0.05). SPT results **(B)**. The sucrose preference of the model group decreased (*p* < 0.01), while both Flu and IRFV treatments increased the sucrose preference (*p* < 0.05 or *p* < 0.01). However, the sucrose preference of IRFV-L also showed no obvious difference (*p* > 0.05). ^##^p < 0.01 vs the control group; *p < 0.05, **p < 0.01 vs the model group.

The immobility time of the TST in all groups is shown in **Figure 2A**. In the model group, the immobility times were dramatically longer than those in the control group (*p* < 0.01). However, after administration of Flu and IRFV, the immobility time shortened significantly (*p* < 0.05 or *p* < 0.01), but no obvious difference was observed in the IRFV-L group (*p* > 0.05). The SPT results are shown in **Figure 2B**. The sucrose preference of the model group decreased (*p* < 0.01), while both Flu and IRFV treatments increased the sucrose preference (*p* < 0.05 or *p* < 0.01). However, the sucrose preference of IRFV-L also showed no obvious difference (*p* > 0.05).

### Effects of the Hippocampal Tissue Contents of NE, 5-HT, and CRF

**Figure 3:**
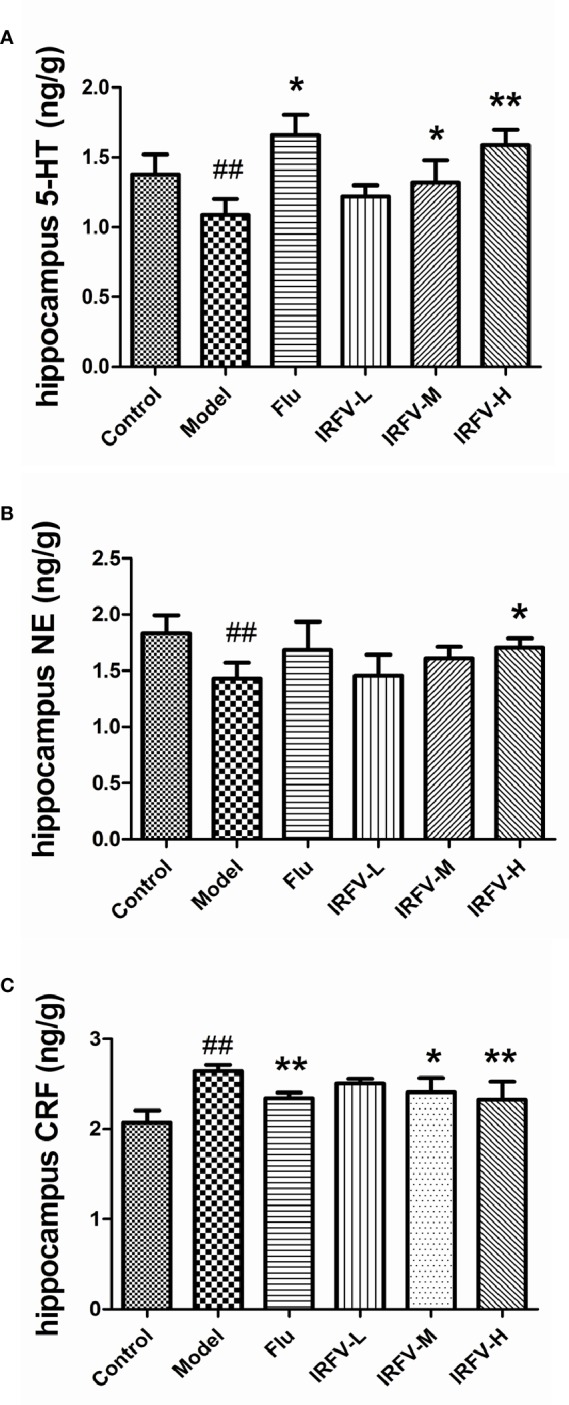
The concentrations of NE and 5-HT were significantly decreased in the model group compared to the control group (*p* < 0.01). When the CUMS mice were treated with Flu and IRFV, their levels of 5-HT observably increased (*p* < 0.01, *p* < 0.05). NA levels significantly increased only in the IRFV-H group (*p* < 0.05). In contrast, CRF increased significantly in the model group compared to the control group (*p* < 0.01). After treatment with Flu and IRFV, the levels of CRF significantly increased compared to the model group (*p* < 0.01, *p* < 0.05), but no obvious difference was observed in the IRFV-L group (*p* > 0.05). ^##^p < 0.01 vs the control group; *p < 0.05, **p < 0.01 vs the model group.

As shown in **Figure 3**, we observed that the concentrations of NE and 5-HT were significantly decreased in the model group compared to the control group (*p* < 0.01). When the CUMS mice were treated with Flu and IRFV, the levels of 5-HT observably increased (*p* < 0.01, *p* < 0.05). NA only significantly increased in the IRFV-H group (*p* < 0.05). In contrast, CRF increased significantly in the model group compared to the control group (*p* < 0.01). After treatment with Flu and IRFV, the levels of CRF significantly increased compared to the model group (*p* < 0.01, *p* < 0.05), but no obvious difference was shown for the IRFV-L group (*p* > 0.05).

### ^1^H NMR Analysis of Serum

**Figure 4:**
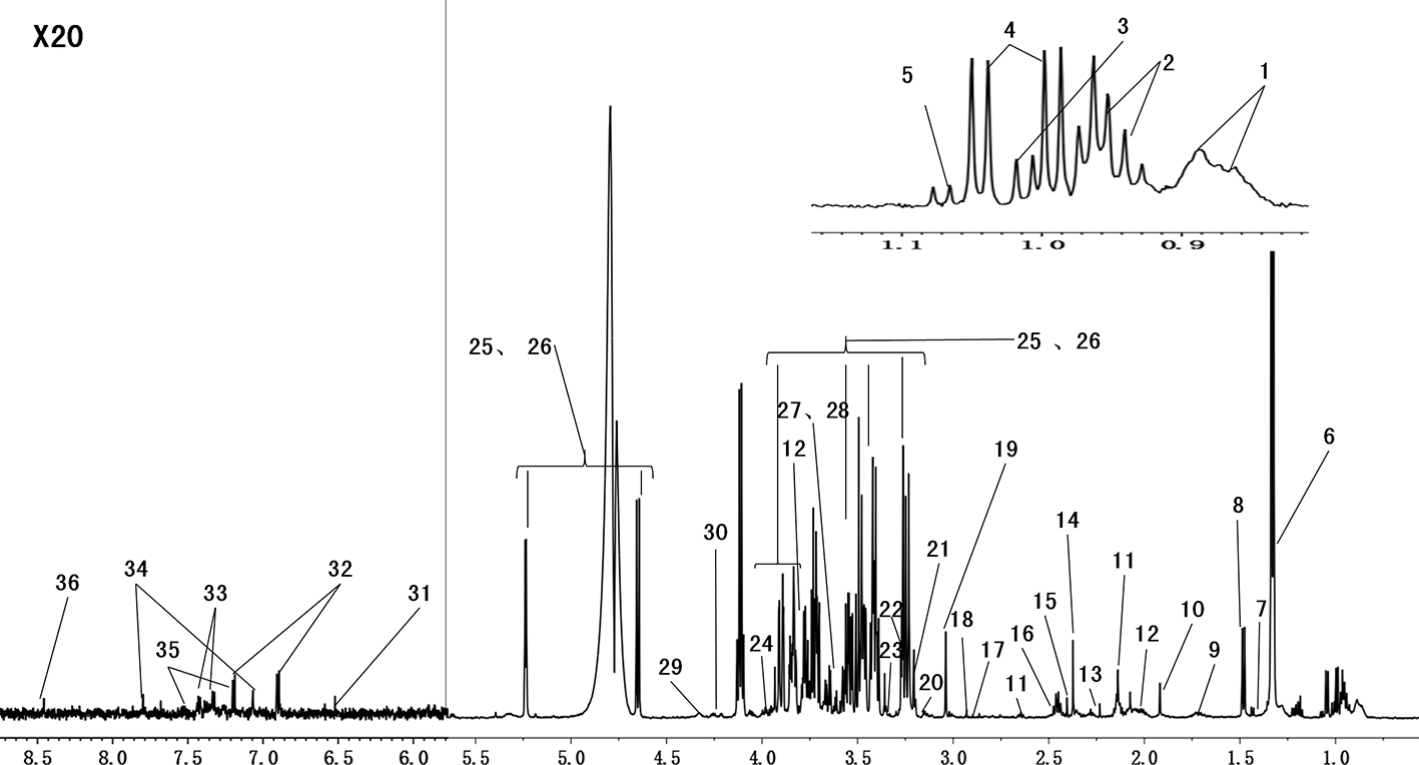
Representative 600 MHz ^1^H-CPMG NMR spectra (*δ* 0.5–4.6, *δ* 5.2–9.0) of serum from the normal groups. The spectra illustrate the majority of the metabolites in this experiment. We marked the metabolites from the relevant peaks of different chemical shifts, which were based on the previous literature and an in-house NMR database. There were 36 metabolites in the serum samples (1, HDL\LDL; 2, isoleucine; 3, leucine; 4, valine; 5, isobutyrate; 6, lactate; 7, lysine; 8, alanine; 9, arginine; 10, acetate; 11, glutamate; 12, glutamine; 13, acetone; 14, pyruvate; 15, succinate; 16, methionine; 17, N,N-dimethylglycine; 18, creatinine; 19, creatine; 20, choline; 21, phosphorylcholine; 22, malate; 23, taurine; 24,betaine; 25, *β*-glucose; 26, *α*-glucose; 27, glycine; 28, glycerol; 29, threonine; 30, myoinositol; 31, fumarate; 32, tyrosine; 33, phenylalanine; 34, histidine; 35, trypotophan; 36, formate).

Serum metabolites may change with CUMS intervention and drug treatment. **Figure 4** shows the representative ^1^H NMR spectra of serum from the control group. The spectra illustrated the majority of the metabolites in this experiment. We marked the metabolites from relevant peaks with different chemical shifts, which were based on previous literature (Tian et al., 2016) and an in-house NMR database, and 36 metabolites were finally identified in the serum samples.

### IRFV Induced Metabolomic Variations in CUMS Mice

**Figure 5:**
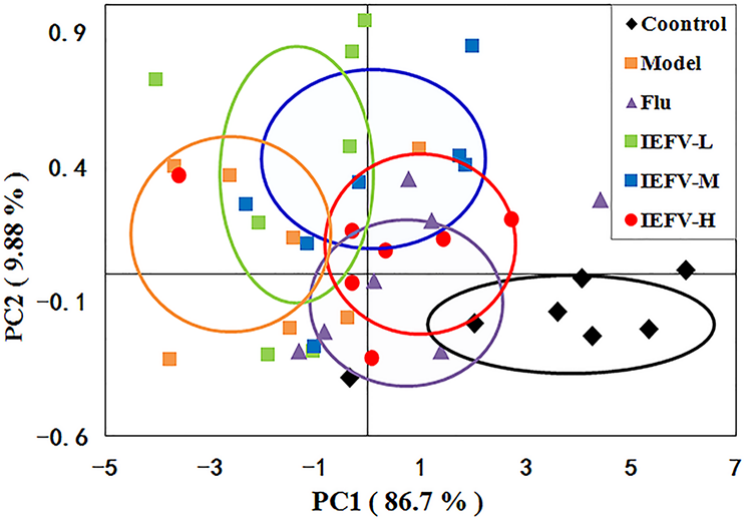
PCA score plots based on the ^1^H NMR spectra of serum samples **(A)** following different dosages of IRFV. In the plot, each dot represents NMR profiling data from an individual sample and colors indicate samples in different treatment groups.

To evaluate the effects of IRFV on the metabolic profiles, we applied unsupervised principal component analysis to explore the separation of the model, Flu, IRFV-L, IRFV-M, IRFV-H, and control groups. As shown in **Figure 5**, the PCA score plot of the serum of the mice was found to be clearly separated among the six groups. The metabolic features of the model group were clearly separated from those of the control and drug-treated groups. Moreover, with increasing doses of IRFV, the metabolic features were closer to those of the control mice. These data indicated a remarkable distinction in the score plots between the model group and the other groups along the PC1 and PC2 axes, with the values of PC1 = 86.7% and PC2 = 9.88% in the serum.

### Metabolic Changes and Biomarker Identification

**Figure 6:**
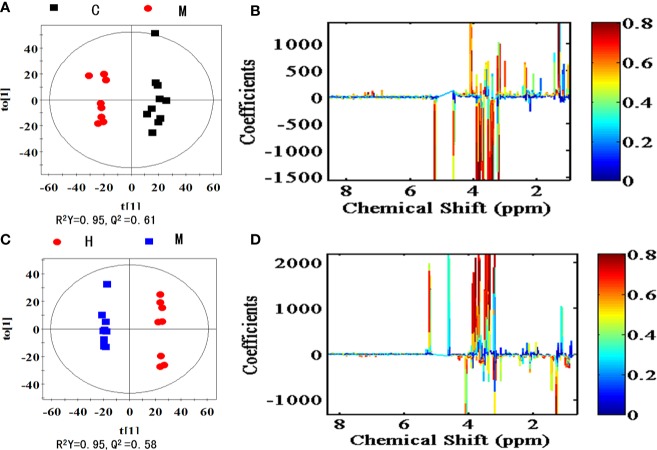
OPLS-DA score plots (left panel) and corresponding coefficient loading plots (right panel) derived from ^1^H NMR spectra of the model group and control group **(A, B)** and the IRFV-H group and model group **(C, D)**. The correlation coefficients are color coded in the coefficient plot, which shows the significance of metabolite variations. Signals with a positive direction indicate that the number of metabolites is higher in the control group than in the model group, and *vice versa*. The metabolites are assigned in **Table 1**.

To explore the metabolites responsible for the separation, OPLS-DA score plots and the corresponding loading plots of serum samples were generated between the model *vs* control group as shown in **Figure 6A** and the IRFV-H *vs* model groups as shown in **Figure 6B**. The comparison of the model and control groups was R^2^Y = 95% and Q^2^Y = 0.61, while the IRFV-H group and model group were R^2^X = 95% and Q^2^Y = 0.58, which presented the explained variance and a high predictive capability, respectively (Westerhuis et al., 2010; Surmacki et al., 2015). The color-coded coefficient plots demonstrating metabolite changes in detail after CUMS intervention are shown in **Figure 6C**, and the IRFV-H-treated results are shown in **Figure 6D**. In this study, potential biomarkers may be identified with VIP values > 1.0 and *p* < 0.05. Compared with the control group, the key biomarkers in the model samples were as follows: decreased levels of fumarate, malate, pyruvate, proline, phenylalanine, arginine, threonine, alanine, lactate, tyrosine, methionine, valine, lysine, isoleucine, myoinositol, N,N-dimethylglycine (DMG), phosphorylcholine, choline, serine, and leucine were found in the model group; and increased levels of glycine, glutamine, betaine, taurine, *α*-glucose and *β*-glucose were found in the model group. The related information (chemical shift, fold change, Components assignment, P value, *etc*.) of the potential metabolites derived from the serum samples is summarized in **Table 1**. With IRFV-H treatment, 21 metabolites returned to normal levels in the serum samples. Additionally, the metabolic biomarkers that were obtained from the serum samples can be summarized as branched amino acids (isoleucine, leucine, valine), other amino acids (tyrosine, glutamate, arginine, *etc*.), organic acids (malate, fumarate, myoinositol), and energy storage compounds (lactate, glucose, fumarate, *etc*.).

**Table 1:** Quantitative comparison of metabolites derived from the ^1^H NMR of serum samples.

Metabolite	Chemical shift (ppm)	Component assignment	FC^a^	VIP score	FC^b^	VIP score
Leucine	0.96(d)	CH_2_	0.88**^#^**	1.39	1.26**	1.56
Valnine	1.03 (d)	CH_3_	0.75**^##^**	1.59	1.62**	1.66
Lactate	1.33 (d)	CH_3_	0.70**^##^**	1.66	1.27**	1.58
Alanine	1.47(d)	CH_3_	0.73**^###^**	1.41	1.51**	1.61
Arginine	1.70 (m)	*δ*-CH_2_	0.75**^#^**	1.52	1.49**	1.62
Lysine	1.74 (m)	*β*-CH_2_	0.75**^##^**	1.37	—	—
glutamate	2.34 (m)	*γ*-CH_2_	0.71**^##^**	1.75	1.37*	1.71
Pyruvate	2.38 (s)	CH_3_	0.84**^#^**	1.38	—	—
Methionine	2.66 (m)	*β*-CH_2_	0.71**^#^**	1.49	—	—
N,N-dimethylglycine	2.94 (s)	CH_3_	0.78**^##^**	1.33	1.32*	1.55
Creatine	3.03(s)	CH3	0.75**^##^**	1.25	—	—
Choline	3.21 (s)	N(CH3)_3_	0.78**^###^**	1.80	1.31*	1.77
Taurine	3.25 (s)	NCH_2_	1.30**^###^**	2.01	0.80**	2.05
β-glucose	3.41 (d)	CH_4_	1.34**^##^**	1.99	0.78**	2.03
Glycine	3.56 (s)	CH_2_	1.17**^###^**	1.66	0.89*	1.51
α-Glucose	3.71 (d)	CH_3_	1.35**^###^**	1.93	0.80**	2.01
phosphorylcholine	3.31 (d)	*β*-CH_3_	0.76**^##^**	1.47	1.48**	1.65
Glutamine	3.77 (t)	*α*-CH	1.19**^##^**	1.82	0.90**	1.66
Betaine	3.91 (s)	N(CH_3_)_3_	1.23**^#^**	1.85	0.84**	1.66
Serine	3.97(m)	*β*-CH	0.85**^#^**	1.21	1.30*	1.57
Myo-inositol	4.06 (t)	H_2_	0.84**^##^**	1.58	—	—
Threonine	4.25(m)	*β*-CH	0.73**^#^**	1.19	1.46**	1.56
Malate	4.31(m)	CH	0.71**^#^**	1.56	1.29*	1.37
Fumarate	6.52(s)	CH	0.14**^##^**	1.65	6.75*	1.51
Tyrosine	6.91 (m)	H_3_/H_5_	0.74**^#^**	1.26	1.45*	1.38
Phenylalanine	7.33 (m)	H_2_/H_6_	0.73**^##^**	1.58	1.69**	1.65

FC^a^: fold change. FC >1 indicates that the amount of metabolites is higher in the model group than in the control group, and vice versa.

FC^b^: fold change. FC >1 indicates that the amount of metabolites is higher in the treated group than in the model group, and vice versa.

compared with control group, ^#^p < 0.05, ^##^p < 0.01, ^###^p < 0.001; compared with model group,*p < 0.05, **p < 0.01.

### Cytoscape to Explore the Biomarker Metabolic Network

**Figure 7:**
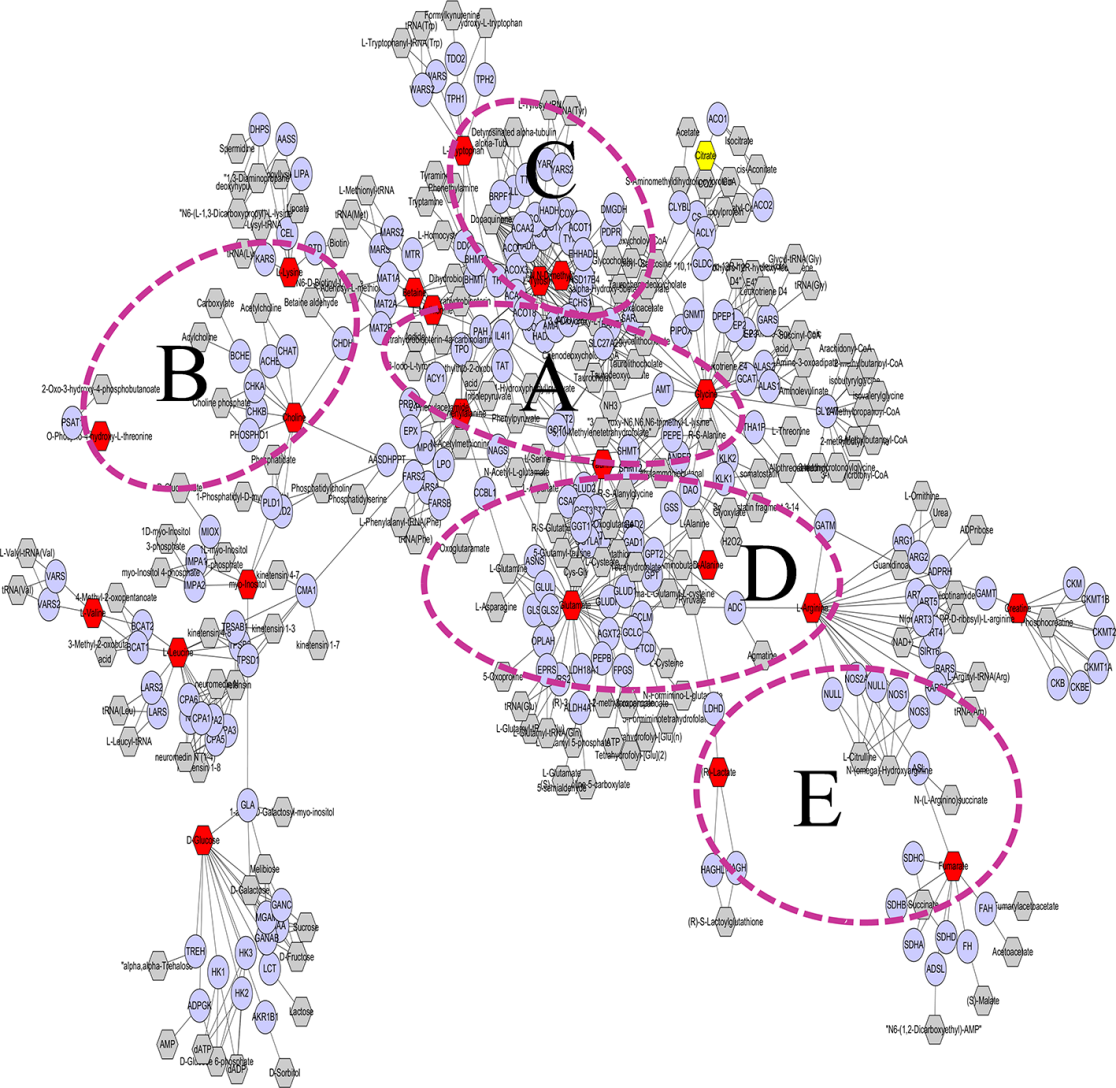
Glycine, serine, methionine, and threonine metabolism are shown **(A)**. Some enzymes and genes were also found to be involved in the TCA cycle and lysine metabolism **(B)** such as phenylalanine hydroxylase (PAH) and tyrosine hydroxylase (TH); tryptophan hydroxylase (TPH) of genes is involved in tryptophan and tyrosine metabolism **(C)**. Glutamic acid decarboxylase (GAD) and gamma-glutamyl transferase (GGT) are involved in arginine, proline, glutamate, aspartate, and asparagine metabolism **(D)**. d-Amino-acid oxidase (DAO), thyroid peroxidase (TPO), and fumarate hydratase (FH) are involved in glycosphingolipid metabolism and methionine and cysteine metabolism **(E)**.

The metabolic networks involved in some enzymes and genes were constructed with Cytoscape to better understand the internal correlation of the potential biomarkers in terms of the enzyme or gene levels (Tian et al., 2018). The metabolic networks that were established based on the markedly different metabolites are shown in **Figure 7**. Glycine, serine, methionine and threonine metabolisms are shown in **Figure 7A**. Some enzymes and genes were also found to be involved in the TCA cycle and lysine metabolism (**Figure 7B**) such as phenylalanine hydroxylase (PAH), tyrosine hydroxylase (TH), and tryptophan hydroxylase (TPH) is involved in tryptophan and tyrosine metabolism (**Figure 7C**). Glutamic acid decarboxylase (GAD) and gamma-glutamyl transferase (GGT) are involved in arginine, proline, glutamate, aspartate and asparagine metabolism (**Figure 7D**). d-Amino-acid oxidase (DAO), thyroid peroxidase (TPO), and fumarate hydratase (FH) are involved in glycosphingolipid, methionine, and cysteine metabolism (**Figure 7E**).

### Metabolic Pathway Analysis

**Figure 8:**
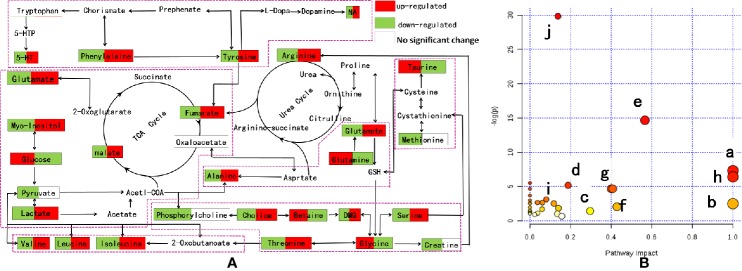
**(A)** Metabolic pathway analysis of IRFV-treated mice from MetaboAnalyst 3.0. The impact is the pathway impact value calculated from pathway topology analysis. Phenylalanine, tyrosine, and tryptophan biosynthesis (a); d-glutamine and d-glutamate metabolism (b); alanine, aspartate, and glutamate metabolism (c); arginine and proline metabolism (d); glycine, serine, and threonine metabolism (e); taurine and hypotaurine metabolism (f); phenylalanine metabolism (g); and valine, leucine, and isoleucine biosynthesis (h). The details of the pathways are shown in panel **B**.

Based on the above biological tests and multiple analyses, potential biomarkers were finally identified in the model and IRFV-treated rats. Subsequently, the relevant metabolic pathways were found using MetaboAnalyst 3.0 (http://www.metaboanalyst.ca/MetaboAnalyst/) (Lauri et al., 2016) and are associated with the series of metabolic responses to the metabolites that were obtained after IRFV treatment. As shown in **Figure 8A**, different points represent different metabolic pathways, and the size of the points represents the impact value that was calculated from the pathway topology analysis. Pathways with an impact value above 0.1 were screened out as potential target pathways. In total, there were eight metabolic pathways involved in this study, including isoleucine, leucine, valine biosynthesis; arginine and proline metabolism; d-glutamine and d-glutamate metabolism; glycine, lysine, threonine, choline, and serine metabolism; the tricarboxylic cycle (TCA cycle); phenylalanine metabolism; tryptophan, phenylalanine, and tyrosine metabolism; and taurine and hypotaurine biosynthesis. The details of the pathways are shown in **Figure 8B**.

## Discussion

Depression affects more than 300 million people worldwide, sometimes leading to a deadly fate in suicide (World Health Organization, 2018). *V. jatamansi* is a famous TCM herb that has been wildly used in Asia and Europe for thousands of years. A previous study from our laboratory reported that IRFV possesses various effects, such as neuroprotective effects, antifree radicals, anti-inflammatory, and even antianxiety (Yan et al., 2010) (Zhang et al., 2018). It may also have antidepressant effects. Thus, the focus of this paper was to investigate the potential effects and mechanisms of IRFV on CUMS-induced depression. A ^1^H NMR-based metabolomic approach was conducted to demonstrate the metabolic differences between normal and CUMS mice with different syndromes in this study and evaluate the effect of IRFV on CUMS mice.

The CUMS model was established to evaluate the activity of antidepressants and has been proven to have high repeatability and consistency with the results of depressive disorders (Raya et al., 2018). The behaviors of the mice after chronic stimulation are consistent with the clinical behaviors of patients with depression. Behavioral tests after CUMS, including the SPT (Klein et al., 2015) and TST (Peng et al., 2007), were applied to investigate the effects of Flu and IRFV. The results demonstrated CUMS hyposensitivity to reward stimulations of sucrose and anhedonia, while the immobility time in the TST reflects the degree of despair. Both Flu and IRFV could relieve the depressive state induced by CUMS interventions, and the effects of IRFV-H were similar to those of Flu.

The CUMS model results in behavioral and physiological abnormalities in animals that may relate to an imbalance of monoamine neurotransmitters in the brain. Our biochemical ﬁndings indicated that IRFV could regulate the levels of monoamine neurotransmitters (5-HT, NA, CRF) and exert antidepressant activity.

### Amino Acid Metabolism and Synthesis of Neurotransmitters

Tyrosine, phenylalanine, and tryptophan have been widely reported in the study of depression models (Eskelund et al., 2016; Keegan et al., 2016). Phenylalanine is an essential amino acid, and its metabolic process is to produce tyrosine with PAH in the liver. Tyrosine can be further metabolized into catecholamine neurotransmitters, such as DA and NE, by TH, which are closely related to depression (Zhao et al., 2015b; Du et al., 2016; Chen et al., 2019). Moreover, tryptophan can be further metabolized to 5-HT by TPH (Pompili et al., 2019). In this study, the concentrations of phenylalanine and tyrosine significantly decreased (McKean et al., 1968; Du et al., 2016; Strasser et al., 2017) in the model group, which may have further affected the synthesis of catecholamine neurotransmitters. We found that the levels of NE and 5-HT significantly decreased in mouse hippocampal tissues, which was consistent with the above result. TH, PAH, and TPH were found in Cytoscape to explore biomarker metabolism, and their enzyme expression was reduced in depression model rats or patients with depression (Guo et al., 2018; Scherer et al., 2018; Lu et al., 2019). This result may indicate that CUMS leads to a decrease in TH, PAH, and TPH activity and that the concentrations of synthetic catecholamine neurotransmitters, tyrosine, and phenylalanine are reduced, resulting in the symptoms of depression.

Glutamate is an important excitatory neurotransmitter of the nervous system in CUMS-induced depression (O'Connor and Cryan, 2013). Interestingly, glutamate and glutamine can interconvert mutually in the body, and glutamine is a precursor of gamma amino butyric acid (GABA) synthesis, which has been found to be deficient in a depression model (Heckers et al., 2002; Ma et al., 2019). Moreover, GAD is the key synthetic enzyme for GABA, which has been found to be deficient in depression models (Heckers et al., 2002; Gao et al., 2013). In addition, glutamine and glutamate can be interconnected through enzyme catalysis, and they are both precursors of glutathione (GSH). As GGT (Yamada et al., 2013) activity decreases, this may influence GSH metabolism. GSH concentrations in the blood serum, plasma, or the brain have also been identified to be significantly decreased in depression (de Souza et al., 2006; Kodydkova et al., 2009; Maes et al., 2011). The glutamate system also regulates HPA axis function, further regulating CRF concentrations in depression (Zafir and Banu, 2009; Davis et al., 2018). In the current study, the level of glutamate signiﬁcantly decreased, and glutamine signiﬁcantly increased in the model group. We also found that the levels of CRF were significantly increased in mouse hippocampal tissues. These results may indicate that CUMS leads to decreased Gad1 and GGT activity and reduced or increased concentrations of CRF, glutamate, and glutamine, resulting in symptoms of depression.

Glycine is biosynthesized in the body from the amino acids serine and threonine. In this study, the levels of glycine, serine, and threonine in the CUMS model group were markedly changed, which may indicate that glycine, serine, and threonine metabolism was disturbed. Changes in glycine, serine, and threonine metabolism have been reported to be associated with depression (Maes et al., 1998; Song et al., 2019). Moreover, glycine is crucial for controlling synaptic plasticity and is an inhibitory neurotransmitter in the central nervous system (Altamura et al., 1995). In addition, glycine is currently a favored therapeutic target for rapid antidepressive action.

Previously reported levels of other amino acids, such as isoleucine, leucine, valine (Jia et al., 2013), arginine, alanine (Hu et al., 2018), threonine, and methionine (Benelli et al., 1999), decreased in the CUMS mice which is consistent with our results. Additionally, isoleucine, leucine, and valine are proteinogenic amino acids with aliphatic side chains and are called branched-chain amino acids (BCAAs). BCAAs can be quickly transported across the blood–brain barrier as major amino group donors for the synthesis of glutamate and 5-HT in the brain (Auer et al., 2000), and the biosynthesis of BCAAs is believed to play a crucial role in the development of depression. Furthermore, we found that arginine and proline metabolism was significantly disturbed, which has been reported in the prefrontal cortex of a learned helplessness rat model in a metabolomics study (Zhou et al., 2017). These amino acid changes may be related to the pathogenesis of CUMS mice.

### Energy Metabolism

Previous studies have indicated that sucrose intake greatly affects alterations in serum metabolites. The tricarboxylic acid (TCA) (Zhao et al., 2015a) cycle is correlated with CUMS-induced depression, and glucose can be metabolized by glycols to produce pyruvate, ATP and lactate, which act as energy substrates. Glycine can also be converted into pyruvate and then into acetyl-coenzyme A (acetyl-CoA), which enters the TCA cycle. The changes in the levels of valine and glycine in the model group indicated that pyruvate metabolism was disturbed (Du et al., 2017). Moreover, fumarate can be further metabolized into L-malate by FH (Mescam et al., 2011). These changes weakened the strength of glycolysis, leading to further energy deficiency. In this experiment, reduced levels of lactate, myoinositol, fumarate, and malate and increased levels of *α*-glucose, *β*-glucose, and glycine indicated that energy production was disturbed in CUMS mice.

### Other Metabolites and Enzymes

Choline can synthesize betaine and phosphatidylcholine, and betaine is further converted into N,N-dimethylglycine (DMG). First, they have an important role in maintaining protein structure and cell membrane integrity. Second, they participate in the body's oxidative stress response (Sussulini et al., 2009). Several previous studies have indicated that depressed patients display a disturbance of gut microﬂora, including metabolites of dimethylglycine and DMG (Zhao et al., 2015d). Moreover, studies have shown that the levels of unsaturated phosphorylcholine, choline, and betaine in depression model animals show an enhanced oxidative effect, which is a typical feature of depression (Liu et al., 2015). It has been reported that choline and betaine change the content of phosphorylcholine and indicate that the integrity of the cell membrane has been damaged after depression, which is consistent with the results of this study (Zhao et al., 2015c). This damage may be related to increased oxidative stress in the body and a decreased immune response (Lever et al., 2017). In our study, the concentrations of choline, phosphorylcholine, betaine, and DMG were obviously changed in CUMS mice. These changes may indicate that oxidative stress destroys the cell membrane structure or gut microﬂora in CUMS mice.

Taurine and myoinositol (Zhang et al., 2009) are the major controllers of osmolarity in the brain. The increasing concentration of taurine and decreasing concentration of myoinositol might disturb osmotic regulation in CUMS mice. Lactate participates in synaptic plasticity, and has been viewed as a good biomarker for brain status (Aalling et al., 2018). Decreased levels of lactate have been found in CUMS mice (Chen et al., 2017). In addition, DAO is a peroxisomal enzyme that is known to oxidize d-serine and may play a regulatory role in N-methyl-d-aspartate-type (NMDA) receptor function (Corvin et al., 2007). The NMDA receptor has a major role in the neurophysiology of depression (Sattar et al., 2018). Moreover, hypothyroidism was determined, and TPO levels were signiﬁcantly decreased with a higher risk of depression, which was expressed during gestation as a marker for subsequent postpartum depression (Dama et al., 2016).

Over all, IRFV downregulated fumarate, malate, phenylalanine, arginine, threonine, alanine, lactate, tyrosine, valine, isoleucine, DMG, choline, serine, and leucine and upregulated glycine, glutamine, betaine, taurine, and glucose in mouse serum. In addition, it is worth noting that whether the activities of some enzymes, such as PAH, TH, TPH, GAD, GGT, DAO, TPO, and FH, were affected by IRFV requires further investigation.

## Conclusions

In this study, a metabolomics approach based on ^1^H NMR was applied to investigate the metabolomics changes in CUMS mouse serum samples. Twenty-six metabolites were identified as biomarkers that were closely related to CUMS mice. Moreover, we paid more attention to the effects of IRFV on CUMS mice. CUMS was used to establish a depressed mouse model, and SPT, TST, 5-HT, NA, and CRF were investigated to evaluate the pharmacodynamic effects. Administration of IRFV could return the behavioral and biochemical indicators to normal and restore metabolic disturbance. Our results suggested that IRFV may protect mice from depression damage *via* regulation of multiple metabolic pathways primarily involving amino acids, energy metabolism and the synthesis of neurotransmitters. These findings provide new perspective insight to better understand the pathophysiological mechanism underlying the CUMS model and are also supported as the basis for follow-up research on the antidepressive mechanism of IRFV.

## Data Availability Statement

All datasets generated for this study are available on request to the corresponding author.

## Ethics Statement

The animal study was reviewed and approved by the Animal Ethical and Welfare of Southwest Jiaotong University (March 19, 2018, No. S20190319002).

## Author Contributions

ZY conceived and designed the experiments. YL, LWu, XZ, TZ, and LWa performed the experiments. CC, CG, YL, and XW analyzed the data. AL guided the experiments. YL wrote the paper. CC revised the paper. All authors read and approved the final manuscript.

## Funding

This work was supported by the Sichuan Province Academic and Technical Leaders Cultivate Support Funds, Key Project of Research and Development Plan of Science and Technology Department of Sichuan Province (Nos. 2018ZR0368 and 2018SZ0078), the Major Scientific and Technological Special Project for “Significant New Drugs Creation” (Nos.2018ZX09201010-001-003, 2019ZX09201005, 2018ZX09735-002) and the National Natural Science Foundation of China (No. 81703948).

## Conflict of Interest

The authors declare that the research was conducted in the absence of any commercial or financial relationships that could be construed as a potential conflict of interest.
